# Triglyceride–Glucose Index and Acute Kidney Injury: A Systematic Review and Meta-Analysis

**DOI:** 10.3390/jcm15093390

**Published:** 2026-04-29

**Authors:** Yan-Wu Yang, Jing Yu, Li Gong, Mei-Ling Ge, Zhi Wan

**Affiliations:** 1 The Emergency Department, West China Hospital, Sichuan University, Chengdu 610041, China; 2 Rare Diseases Center, West China Hospital, Sichuan University, Chengdu 610041, China; 3 Center of Gerontology and Geriatrics, National Clinical Research Center for Geriatrics, West China Hospital, Sichuan University, Chengdu 610041, China

**Keywords:** triglyceride–glucose index, acute kidney injury, insulin resistance, cardiovascular disease, meta-analysis

## Abstract

**Background:** The triglyceride–glucose (TyG) index, a surrogate marker of insulin resistance, has been increasingly associated with adverse cardiometabolic outcomes. However, its relationship with acute kidney injury (AKI) across clinical settings has not been comprehensively synthesized. We performed a systematic review and meta-analysis to evaluate the association between the TyG index and AKI risk. **Methods:** PubMed, Embase, and Web of Science were searched through February 2026 for observational studies reporting adjusted associations between TyG and AKI. Random-effects models were used to pool categorical and continuous effect estimates. Dose–response analyses and subgroup assessments were conducted to explore consistency. Diagnostic performance was summarized when available. **Results:** Thirty studies involving 518,677 participants were included. Compared with the lowest-TyG category, the highest-TyG category was associated with significantly increased AKI risk (pooled OR = 1.95, 95% CI 1.66–2.28; I^2^ = 72.7%). Associations were most consistently observed in cardiovascular disease cohorts (pooled OR = 1.86, 95% CI 1.44–2.40) and remained directionally similar across other clinical settings without significant subgroup interactions. Each 1-unit increment in TyG index was associated with higher AKI risk (OR = 1.48, 95% CI 1.30–1.69), and time-to-event analyses yielded concordant results (HR = 1.38, 95% CI 1.14–1.65). A significant non-linear dose–response association was observed (*p* < 0.001). The TyG index showed moderate discrimination (AUC = 0.74), with findings remaining robust in sensitivity analyses. **Conclusions:** Current evidence supports TyG primarily as a complementary metabolic risk marker rather than a substitute for established AKI diagnostic or prediction tools. TyG may serve as a practical marker for metabolic risk stratification in patients at risk of AKI.

## 1. Introduction

Insulin resistance represents a central component of metabolic dysregulation and is closely linked to endothelial dysfunction, low-grade inflammation, oxidative stress, and microvascular impairment [1]. These disturbances contribute not only to the development of atherosclerotic cardiovascular disease but may also increase susceptibility to acute organ injury under physiological stress [1,2]. As metabolic and cardiovascular disorders continue to rise worldwide, identifying simple and accessible markers that reflect systemic vulnerability has become increasingly relevant for clinical risk stratification [2,3].

The triglyceride–glucose (TyG) index, derived from fasting triglyceride and glucose concentrations, has emerged as a practical surrogate marker of insulin resistance, with high sensitivity and specificity compared with the hyperinsulinemic–euglycemic clamp [2]. Owing to its availability in routine laboratory testing and reproducibility across populations, the TyG index and its derivatives have been widely studied in relation to cardiovascular outcomes, including incident heart failure, coronary artery disease, stroke, cardiovascular–kidney–metabolic (CKM) syndrome, and mortality [2,4,5,6]. Elevated TyG levels have consistently been associated with adverse vascular events and death, and improve risk discrimination beyond traditional factors, suggesting that this index captures aspects of metabolic and endothelial dysfunction [1,2,4,6,7].

However, whether this metabolic signal translates into an increased risk of acute kidney injury (AKI) has not been comprehensively evaluated. AKI is a frequent and clinically significant complication in patients with cardiovascular disease and in those experiencing acute systemic illness, such as acute coronary syndrome, cardiac surgery, sepsis, and intensive care unit (ICU) admission, and even transient renal dysfunction is associated with progression to chronic kidney disease and excess cardiovascular risk [2,8]. Recent observational studies in high-risk settings, including contrast-induced AKI and critically ill patients with heart failure, suggest a positive association between a higher TyG index and AKI incidence or worse renal outcomes, but findings remain heterogeneous and largely single-center [8,9,10]. Differences in study populations, exposure modeling, and analytical strategies limit comparability, and it is unclear whether the association is consistent across cardiovascular and systemic illness contexts, follows a dose–response pattern, or is modified by baseline characteristics.

Given the central role of insulin resistance in CKM pathophysiology and its impact on renal hemodynamics, neurohormonal activation, inflammation, and oxidative stress [1,6,8], the TyG index may represent an accessible indicator of systemic vulnerability to AKI. In addition, the potential clinical value of TyG-based metrics for risk stratification has been demonstrated in multiple cardiovascular and kidney settings but not systematically quantified for AKI. We therefore conducted a systematic review and meta-analysis to evaluate the association between the TyG index and AKI risk across cardiovascular and systemic illness settings. We separately synthesized categorical and continuous effect estimates, assessed dose–response relationships, examined potential effect modification, and summarized available evidence on diagnostic performance, aiming to clarify the strength of the association, potential sources of heterogeneity, and the possible role of TyG as a complementary metabolic risk marker in AKI.

## 2. Methods

This systematic review and meta-analysis were conducted in accordance with the PRISMA 2020 statement. A completed PRISMA checklist is provided in the Supplementary Materials Table S1.

### 2.1. Literature Search

A comprehensive literature search was performed in PubMed, Embase (Ovid), and Web of Science from database inception to February 2026. Search terms combined concepts related to the triglyceride–glucose (TyG) index and acute kidney injury (AKI). Exposure-related keywords included “triglyceride-glucose index”, “TyG”, and related expressions. Outcome-related terms included “acute kidney injury”, “AKI”, “contrast-induced AKI”, and “cardiac surgery–associated AKI”. Search strategies were adapted to the syntax and indexing structure of each database. No restrictions were imposed regarding study population or clinical setting at the database search stage; eligibility restrictions were applied during study screening and selection. The detailed search strategies are provided in the Supplementary Materials. Reference lists of relevant articles and reviews were also screened manually to identify additional eligible studies. Conference abstracts, editorials, case reports, reviews, and non-original studies were excluded.

### 2.2. Study Selection

Studies were considered eligible if they enrolled adult participants, evaluated the TyG index as the exposure of interest, and reported AKI as an outcome defined according to established criteria such as the Kidney-Disease: Improving Global Outcomes (KDIGO) or comparable definitions. Eligible studies were required to provide effect estimates, including odds ratios or hazard ratios with corresponding 95% confidence intervals (95% CI), for the association between TyG index and AKI. Both categorical comparisons and continuous analyses were included. Only original observational studies published in peer-reviewed journals were considered.

Studies were excluded if they did not report AKI outcomes, did not evaluate the TyG index, or lacked sufficient data to extract effect estimates. When multiple reports were derived from the same database, study periods and inclusion criteria were carefully compared to minimize potential overlap.

### 2.3. Data Extraction

Study screening and data extraction were performed independently by two investigators (YWY and MLG). Disagreements were resolved by consensus. For each study, we extracted information on author, publication year, country, study design, population characteristics, sample size, number of AKI events, mean or median age, sex distribution, prevalence of diabetes where available, and the definition of AKI used. Details of the exposure definition, including categorical comparisons and continuous modeling approaches, were recorded. The most fully adjusted effect estimates and corresponding confidence intervals were extracted whenever available, along with the covariates included in multivariable models.

### 2.4. Quality and Publication Bias Assessment

Risk of bias was independently assessed by two reviewers (YWY and MLG) using the Quality in Prognostic Studies (QUIPS) tool [11]. The QUIPS framework evaluates six domains: study participation, study attrition, prognostic factor measurement, outcome measurement, study confounding, and statistical analysis and reporting. Each domain was judged as having a low, moderate, or high risk of bias. Disagreements were resolved by consensus.

### 2.5. Statistical Analysis

Odds ratios (OR) and hazard ratios (HR) were synthesized separately using random-effects models. For categorical analyses, estimates comparing the highest versus lowest TyG category were preferentially pooled, while continuous associations (per-unit increment) were analyzed independently. Effect sizes were log-transformed before pooling. Between-study heterogeneity was quantified using I^2^ and τ^2^. Pre-specified subgroup analyses were conducted according to age, sex, diabetes, hypertension, chronic kidney disease, and disease category, with subgroup differences evaluated using ratio-of-odds-ratios. Dose–response relationships were assessed using a two-stage random-effects approach with restricted cubic splines to evaluate non-linearity. Study-level moderators were examined using random-effects meta-regression, and the proportion of heterogeneity explained was estimated using the R^2^ analog. Diagnostic performance was summarized using bivariate random-effects models. Sensitivity analyses were performed using leave-one-out procedures, and publication bias was assessed using funnel plots and Egger’s test when applicable.

All statistical analyses were performed using R software (version 4.4.2; R Foundation for Statistical Computing, Vienna, Austria), primarily with the meta, metafor, dosresmeta, and mada packages where appropriate.

## 3. Results

**Figure 1:**
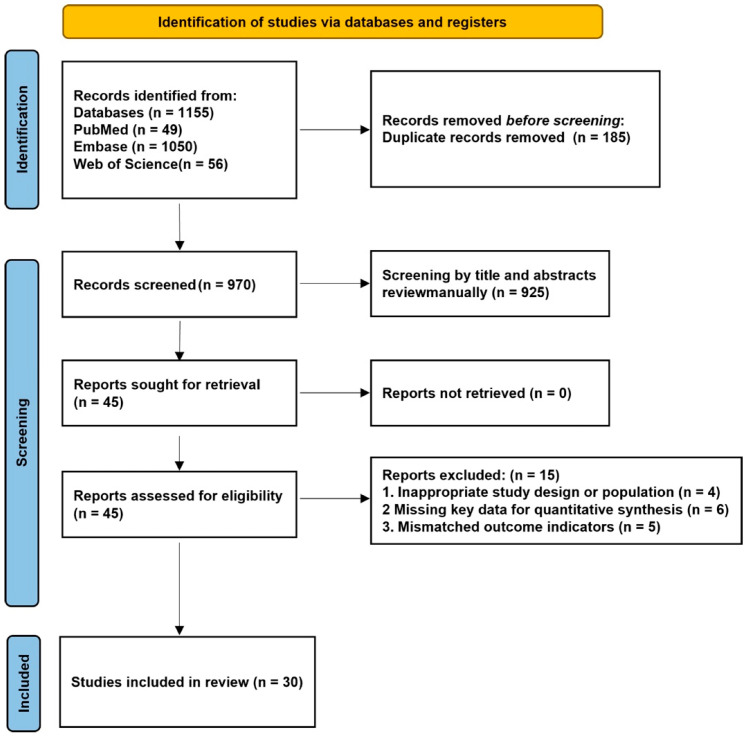
PRISMA flow diagram of study selection. A total of 30 observational studies involving 518,677 participants were included in the meta-analysis (Table 1). Sample sizes ranged from 110 to 427,659 individuals. The majority of studies were retrospective in design (*n* = 28), with two prospective cohorts. All studies were conducted in China and applied KDIGO criteria for AKI definition, except one using European Society of Urogenital Radiology (ESUR) criteria for contrast-induced AKI. The included populations were clinically heterogeneous, encompassing cardiovascular diseases (acute myocardial infarction (AMI), coronary artery disease (CAD), percutaneous coronary intervention (PCI), coronary artery bypass grafting (CABG)), sepsis, traumatic brain injury, pancreatitis, intracerebral hemorrhage, ICU cohorts, and community-based populations. Mean age across studies ranged from 36.8 to 71.7 years, and the proportion of male participants varied between 34.9% and 81.1%. The prevalence of diabetes ranged from 5.6% to 100%, depending on the underlying cohort. TyG index was analyzed predominantly as a continuous exposure, frequently complemented by categorical analyses based on quartiles or quintiles. AKI incidence varied substantially across clinical settings, ranging from 4.7% in subarachnoid hemorrhage to over 80% in heart failure and sepsis cohorts, reflecting differences in baseline risk and disease severity. Methodological quality was assessed using the QUIPS tool. Among the included studies, twenty-two were rated as having an overall low risk of bias, while eight were judged to have a moderate risk of bias. Detailed domain-specific quality assessments were presented in Supplementary Table S3.

The literature search across PubMed, Embase, and Web of Science yielded a total of 1155 records. After removing 185 duplicates, 970 unique articles were screened by title and abstract. Following preliminary screening, 45 studies were retrieved for full-text review. Of these, 15 were excluded due to ineligible populations or study designs, missing key data required for quantitative synthesis, or outcomes not relevant to acute kidney injury. Ultimately, 30 studies fulfilled all inclusion criteria and were incorporated into the final meta-analysis [8,12,13,14,15,16,17,18,19,20,21,22,23,24,25,26,27,28,29,30,31,32,33,34,35,36,37,38,39,40]. The detailed screening process is summarized in Figure 1, following the PRISMA 2020 guidelines, with comprehensive search strategies provided in Supplementary Materials: Table S2.

**Table 1:** Characteristics of the studies included in the meta-analysis.

Study	Design	Population	N	AKI (%)	Mean Age (years)	Male (%)	DM (%)	Exposure Model	AKI Definition
Qin 2020 [25]	Prospective	T2DM with CAD	928	21.2	68	71	100	Quartiles	KDIGO
Hu 2022 [17]	Retrospective	PCI	1073	19.9	NR	67	100	Quartiles	KDIGO
Yang 2023 [8]	Retrospective	Heart failure	1393	82.8	71	59	37.6	Per 1-unit; Quartiles	KDIGO
Jin 2023 [21]	Retrospective	ICU patients	54,263	25.1	59	48	8.9	Per 1-unit; Quartiles	KDIGO
Zhu 2023 [40]	Retrospective	T2DM undergoing PCI	722	9.6	64	68	100	Binary	ESUR CI-AKI
Jin 2024 [20]	Retrospective	AMI	1101	64.7	65	65	28.3	Per 1-unit; Quartiles	KDIGO
Fang 2024 [14]	Retrospective	Sepsis	1426	78.5	62	56	28.6	Per 1-unit; Quartiles	KDIGO
Huang 2024 [18]	Retrospective	Traumatic brain injury	492	59.3	69	56	26.2	Per 1-unit; Quartiles	KDIGO
Cai 2024 [12]	Retrospective	AMI	1831	15.6	64	69	14.8	Per 1-unit; Quartiles	KDIGO
Shi 2024 [27]	Retrospective	Coronary revascularization	790	30.1	68	72	15.8	Per 1-unit; Quartiles	KDIGO
Wang 2025 [30]	Retrospective	Acute pancreatitis	848	61.6	57	59	17.6	Per 1-unit	KDIGO
Chi 2024 [13]	Prospective	Acute pancreatitis	258	30.6	62	75	63.6	Binary	KDIGO
He 2024 [15]	Retrospective	Hyperlipidemic pancreatitis	110	20.9	37	72	NR	Binary	KDIGO
Wang 2024 [29]	Retrospective	AECOPD	645	40.0	67	49	28.4	Per 1-unit; Quartiles	KDIGO
Zhang 2024 [35]	Retrospective	Hypertensive patients	4418	56.1	67	57	NR	Per 1-unit; Quartiles	KDIGO
Zhou 2025 [39]	Retrospective	Community cohort	427,659	5.3	57	46	9.2	Per 1-SD; Quintiles	KDIGO
Zhang 2025 [37]	Retrospective	CAD	1501	67.2	70	62	33.0	Per 1-unit; Quartiles	KDIGO
Zhang 2025 [34]	Retrospective	Sepsis	2616	78.0	NR	57	NR	Per 1-unit; Quartiles	KDIGO
Pan 2025 [24]	Retrospective	Traumatic brain injury	1505	9.4	55	66	22.4	Per 1-unit; Binary	KDIGO
Qiu 2025 [26]	Retrospective	Subarachnoid hemorrhage	3271	4.7	55	35	5.6	Per 1-unit; Quartiles	KDIGO
Hou 2025 [16]	Retrospective	CABG	3260	15.8	64	74	40.0	Per 1-unit; Quartiles	KDIGO
Li 2025 [22]	Retrospective	CABG	296	44.6	67	81	36.0	Binary	KDIGO
Yang 2025 [31]	Retrospective	CAD	2517	NR	69	68	38.8	Per 1-unit; Quartiles	KDIGO
Zhang 2025 [38]	Retrospective	Cardiac surgery	542	55.7	67	60	19.7	Per 1-unit; Quartiles	KDIGO
Zhang 2025 [36]	Retrospective	AMI	1227	71.5	67	69	31.0	Per 1-unit	KDIGO
Jin 2025 [19]	Retrospective	Diabetic ketoacidosis	678	28.9	47	53	100	Quartiles	KDIGO
Wang 2025 [28]	Retrospective	Sepsis	997	75.0	67	41	NR	Quartiles	KDIGO
Lu 2025 [23]	Retrospective	Atrial fibrillation	1142	42.1	72	55	27.2	Per 1-unit; Quartiles	KDIGO
Zhang 2025 [33]	Retrospective	PCI	435	25.7	67	67	31.7	Per 1-unit	KDIGO
Zeng 2025 [32]	Retrospective	Intracerebral hemorrhage	733	15.6	70	54	NR	Binary	KDIGO

T2DM, type 2 diabetes mellitus; CAD, coronary artery disease; PCI, percutaneous coronary intervention; CABG, coronary artery bypass grafting; ICU, intensive care unit; AECOPD, acute exacerbation of chronic obstructive pulmonary disease; AMI, acute myocardial infarction; AKI, acute kidney injury; KDIGO, Kidney Disease: Improving Global Outcomes; ESUR, European Society of Urogenital Radiology; CI-AKI, contrast-induced acute kidney injury; NR, not reported; SD, standard deviation.

## 4. Association Between TyG Index and AKI Risk

### 4.1. Categorical Analyses

**Figure 2:**
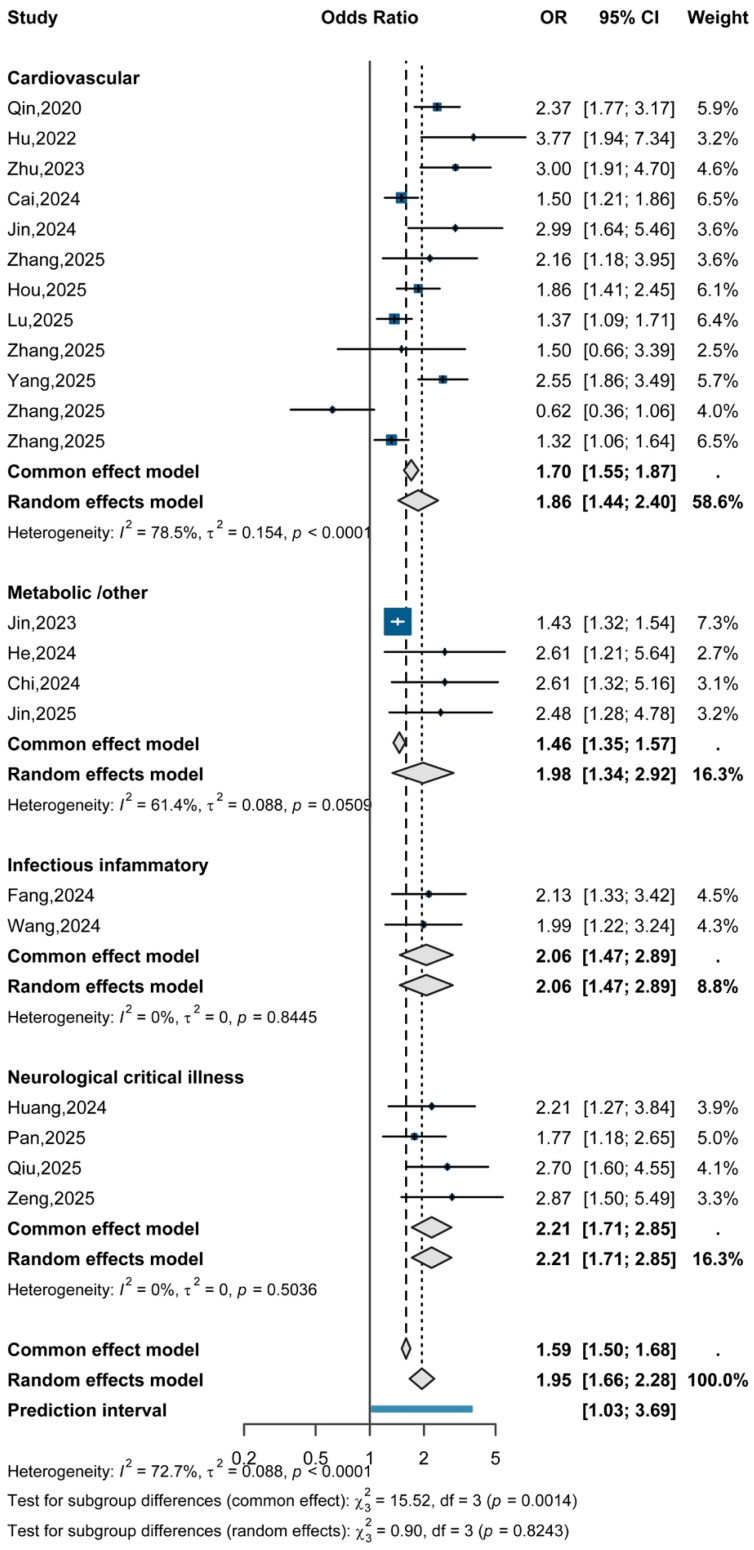
Association between TyG index and AKI risk across different clinical populations.

A total of 22 studies were included in the categorical synthesis assessing the association between the TyG index and AKI risk. Comparing the highest versus lowest TyG categories, elevated TyG levels were significantly associated with increased AKI risk (random-effects OR = 1.95, 95% CI: 1.66–2.28), with substantial between-study heterogeneity (I^2^ = 72.7%) (Figure 2).

Stratified analyses by underlying disease category showed that the direction of association was generally positive across clinical contexts; however, substantial between-study heterogeneity remained, and these findings should not be interpreted as evidence of uniform effects across populations. The pooled OR was 1.86 (95% CI: 1.44–2.40) in cardiovascular diseases, 1.98 (95% CI: 1.34–2.92) in metabolic-related conditions (acute pancreatitis, diabetic ketoacidosis, and ICU cohorts), 2.06 (95% CI: 1.47–2.89) in infectious or inflammatory settings (sepsis, AECOPD), and 2.21 (95% CI: 1.71–2.85) in neurological critical illness cohorts (intracerebral hemorrhage, traumatic brain injury, and subarachnoid hemorrhage). Tests for subgroup differences did not indicate statistically significant variation across these disease categories.

### 4.2. Continuous Analyses

**Figure 3:**
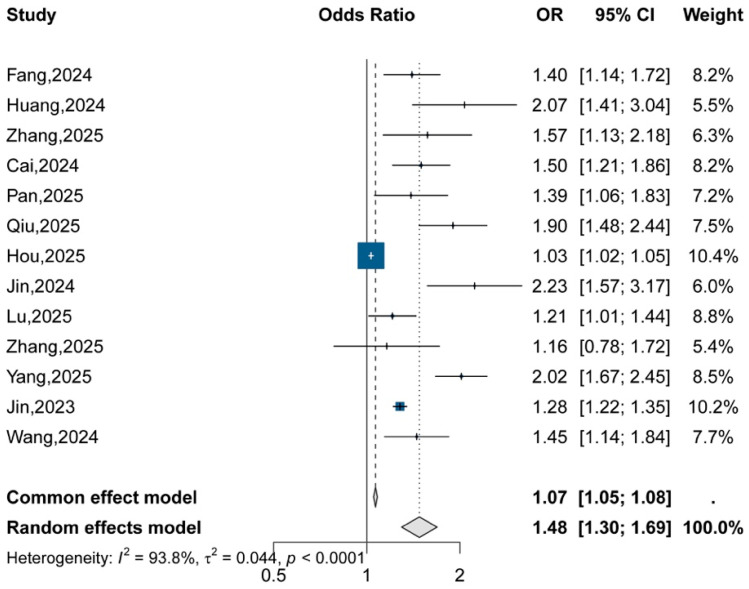
Forest plot of pooled odds ratios for AKI per 1-unit increase in TyG index.

Given the considerable heterogeneity, this pooled estimate should be interpreted cautiously and viewed primarily as an overall summary of direction rather than a uniformly transportable effect magnitude. When the TyG index was analyzed as a continuous variable, each 1-unit increment was associated with higher odds of AKI (random-effects OR = 1.48, 95% CI: 1.30–1.69), although heterogeneity was considerable (I^2^ = 93.8%) (Figure 3). These findings indicate that the association persisted across different exposure parameterizations.

### 4.3. Hazard Ratio–Based Analyses

**Figure 4:**
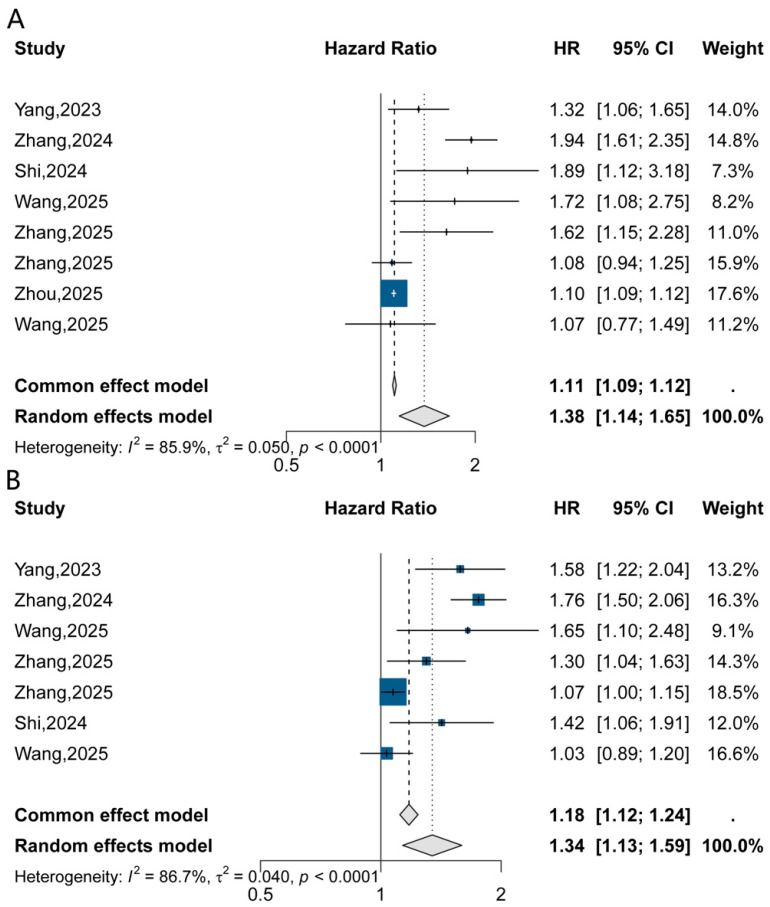
Hazard ratio–based analyses of TyG index and AKI risk. (**A**) Hazard ratios for AKI risk associated with higher TyG levels based on categorical comparisons across included studies. (**B**) Hazard ratios for AKI risk per unit increase in TyG index based on continuous models.

Time-to-event analyses yielded directionally consistent results. In categorical models, an elevated TyG index was associated with increased AKI risk under random-effects modeling (pooled HR = 1.38, 95% CI: 1.14–1.65), with substantial heterogeneity observed (I^2^ = 85.9%) (Figure 4A). Continuous HR-based analyses demonstrated comparable effect direction and magnitude (Figure 4B).

### 4.4. Subgroup and Interaction Analyses

**Figure 5:**
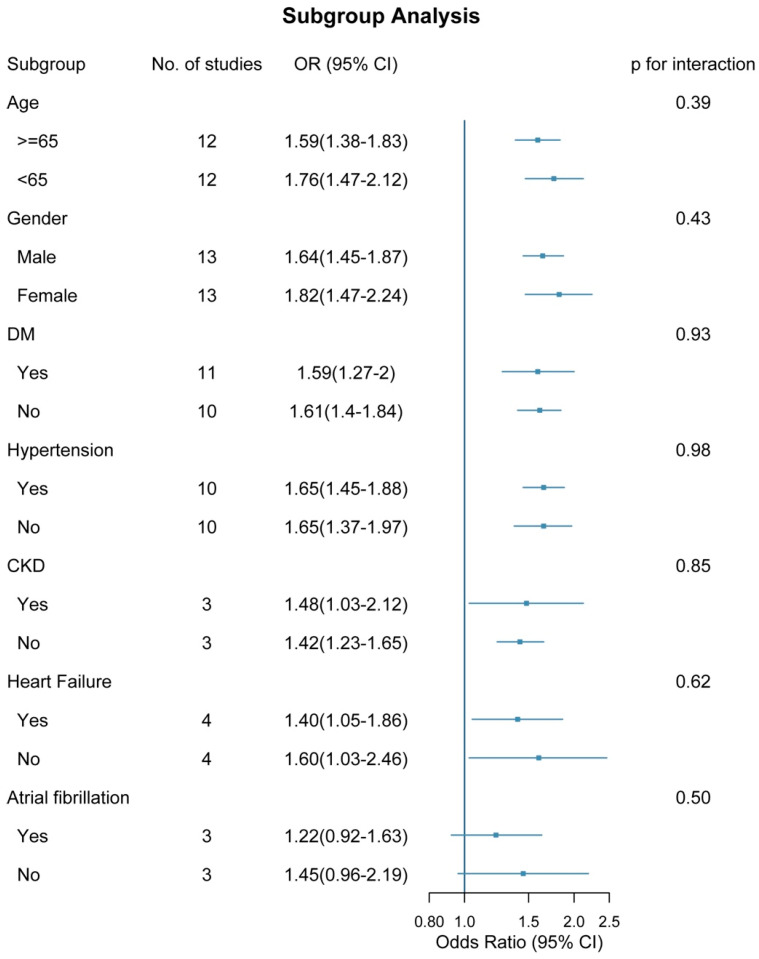
Subgroup effects of TyG index on AKI risk.

Subgroup analyses showed that the positive association between the TyG index and AKI was generally consistent across strata defined by age, sex, diabetes, hypertension, chronic kidney disease, heart failure, and atrial fibrillation. Pooled effect estimates were directionally similar across subgroups, and most remained statistically significant. Formal tests for interaction provided no evidence of effect modification (all *p* for interaction >0.05 Figure 5). Consistently, ratio-of-odds-ratios meta-analyses yielded pooled interaction estimates centered around unity, further supporting the robustness and homogeneity of the observed association (Figure S2).

### 4.5. Meta-Regression

To explore potential sources of between-study heterogeneity, univariable random-effects meta-regression analyses were performed using study-level characteristics. Among the examined moderators, diabetes prevalence was positively associated with effect size, with each 1% increase in diabetes proportion corresponding to a 1% increase in the pooled odds ratio (β = 0.006, 95% CI: 0.001–0.012; *p* = 0.021), explaining approximately 50% of between-study variance. In contrast, mean age (*p* = 0.194), male proportion (*p* = 0.622), publication year (*p* = 0.168), study design (*p* = 0.289), and disease category (all *p* > 0.300) were not significantly associated with effect size (Table S4). These findings suggest that underlying diabetes burden at the study level may partially account for heterogeneity in the association between TyG index and AKI risk, whereas demographic characteristics and study design do not materially modify the observed effect. Meta-regression suggested that study-level diabetes prevalence was associated with variation in effect size and may explain part of the observed between-study heterogeneity. However, this finding should be interpreted cautiously because it was not paralleled by statistically significant subgroup interaction results.

### 4.6. Diagnostic Accuracy and Summary ROC Analysis

**Figure 6:**
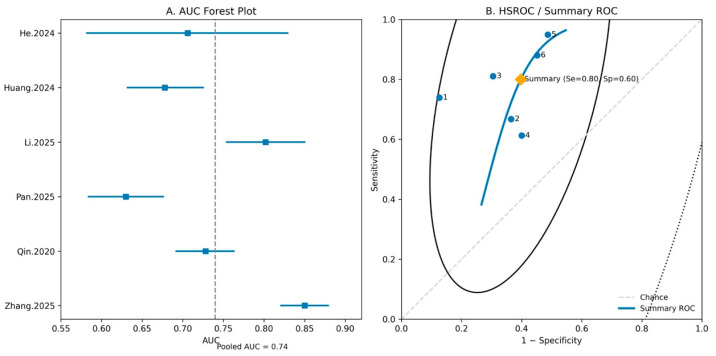
Diagnostic performance of the TyG index for predicting AKI.

A diagnostic accuracy meta-analysis including six studies was conducted to evaluate the discriminative performance of the TyG index for predicting acute kidney injury. The pooled sensitivity was 0.81 (95% CI: 0.67–0.89) and the pooled specificity was 0.64 (95% CI: 0.56–0.71). The pooled positive likelihood ratio was 2.05 (95% CI: 1.75–2.41), and the pooled negative likelihood ratio was 0.30 (95% CI: 0.18–0.49), corresponding to a pooled diagnostic odds ratio of 7.53 (95% CI: 3.96–14.32). Figure 6 presents the forest plot of study-specific AUCs and the summary receiver operating characteristic (ROC) curve. The pooled AUC was 0.74 (95% CI, 0.66–0.81), indicating moderate overall discriminative performance across the included studies.

### 4.7. Non-Linear Dose–Response Relationship Between TyG Index and AKI Risk

**Figure 7:**
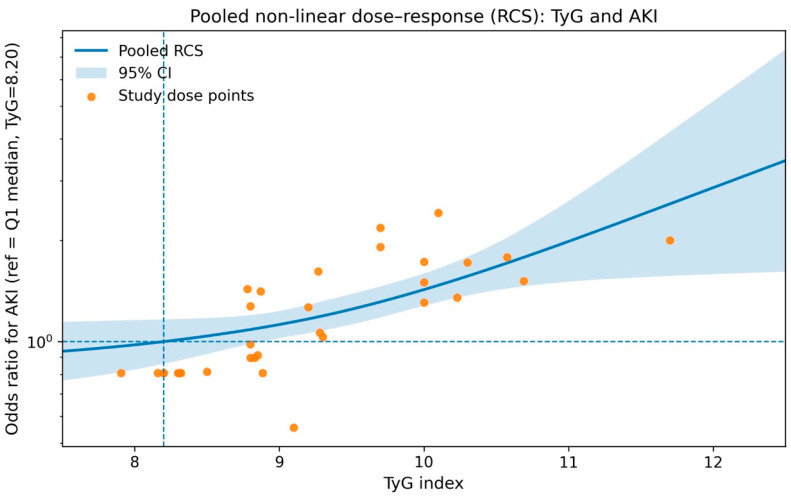
Pooled non-linear dose–response relationship between TyG index and AKI risk.

In the non-linear dose–response meta-analysis including nine studies, a significant non-linear association between the TyG index and the risk of acute kidney injury was observed (*p* for non-linearity <0.001 Figure 7). Using the median TyG value of the lowest exposure category (TyG = 8.20) as the reference, the pooled restricted cubic spline curve demonstrated a relatively flat risk profile at lower TyG levels, followed by a progressively steeper increase in AKI risk with increasing TyG index. Notably, the risk of AKI rose monotonically beyond approximately TyG 8.5–9.0, with a marked elevation at higher TyG values. Confidence intervals widened at the lower end of the TyG distribution, reflecting limited data in this range; however, the overall pattern consistently supported a dose-dependent increase in AKI susceptibility associated with higher TyG levels.

### 4.8. Publication Bias and Sensitivity Analyses

Publication bias was assessed using funnel plots and Egger’s regression test. In the overall analysis (22 studies), funnel plot asymmetry was observed and confirmed by a significant Egger’s test (t = 3.64, df = 20, *p* = 0.002), indicating potential publication bias. Trim-and-fill analysis imputed nine missing studies and yielded an adjusted pooled OR of 1.55 (95% CI: 1.29–1.87). Compared with the primary random-effects pooled estimate of 1.95 (95% CI: 1.66–2.28), this represents a meaningful attenuation, although the association remained statistically significant after adjustment (Figure S3). In cardiovascular cohorts (12 studies), the funnel plot was symmetric and Egger’s test was not significant (t = 1.20, df = 10, *p* = 0.257), suggesting low risk of publication bias in this subgroup (Figure S4). Influence analysis identified one potentially influential study [34]; however, exclusion of this study did not materially alter the results (pooled OR 2.01 vs. 1.95), and heterogeneity decreased (τ^2^: 0.088 to 0.057), supporting the robustness of the findings (Figure S5).

## 5. Discussion

In this systematic review and meta-analysis, an elevated TyG index was associated with an increased risk of AKI across diverse clinical settings, although substantial heterogeneity was observed, particularly in the overall categorical and continuous analyses. The association remained statistically significant in both categorical and continuous models and demonstrated a non-linear dose–response pattern. The evidence appeared most robust in cardiovascular cohorts, including patients with CAD, myocardial infarction, and those undergoing revascularization procedures, where the number of studies was greatest and the pooled estimates were relatively more stable. Subgroup and interaction analyses did not identify statistically significant differences across several study-level characteristics; however, these findings should not be interpreted as evidence of uniform effects across populations. Although discriminative performance was moderate, the current evidence supports TyG more appropriately as a candidate marker of metabolic vulnerability than as a validated clinical tool for AKI risk stratification.

The observed pattern can be interpreted within a metabolic–vascular–renal framework. The TyG index reflects insulin resistance, a core feature of cardiometabolic dysregulation that promotes endothelial dysfunction, oxidative stress, and microvascular damage, thereby linking metabolic and vascular injury pathways [41,42,43]. In cardiovascular disease, chronic exposure to hyperglycemia, hypertriglyceridemia, and neurohormonal activation fosters structural and functional microcirculatory changes that impair renal autoregulation and reserve [42,44,45]. Under perioperative or critical illness conditions, such as revascularization procedures or intensive care admission, these pre-existing abnormalities may amplify the impact of hemodynamic instability, nephrotoxic exposure, and systemic inflammation on renal perfusion and tubular integrity, lowering the threshold for AKI [44,45,46]. Consistent with this concept, TyG has been associated with incident kidney dysfunction and AKI across contrast-induced, heart failure, and other high-risk settings, and with adverse renal outcomes such as need for renal replacement therapy [8,47]. The relative robustness of TyG–AKI associations in coronary and revascularization cohorts is therefore compatible with a pathophysiological milieu in which insulin resistance, endothelial dysfunction, and renal microvascular vulnerability converge, and in which TyG serves as an integrated marker of this systemic susceptibility [41,42,46].

Beyond vascular dysfunction alone, these findings also support the concept of cardiorenal–metabolic susceptibility to renal injury. In meta-regression analyses, higher proportions of diabetes were associated with larger TyG–AKI effect estimates, in line with evidence that diabetes and metabolic syndrome markedly increase AKI incidence and worsen its long-term renal and cardiovascular consequences [48,49]. This gradient is biologically plausible. Chronic insulin resistance promotes glomerular hyperfiltration, intraglomerular hypertension, lipid accumulation in renal tissue, endothelial activation, and low-grade inflammation, all of which reduce renal reserve and predispose to maladaptive repair after injury [8,45,50,51]. Experimental and clinical data further show that insulin resistance activates sympathetic and renin–angiotensin–aldosterone pathways, enhances sodium retention, and aggravates cardiorenal congestion, thereby amplifying susceptibility to ischemia, contrast exposure, surgery, or systemic stress [45,50,52]. Importantly, however, the association between TyG and AKI was not confined to diabetic populations. Subgroup and interaction analyses showed no statistically significant effect modification by diabetes status, consistent with observations that insulin resistance and TyG-based indices predict progression across cardio-renal-metabolic disease states, including CKD, even in non-diabetic individuals [8,53,54]. TyG thus appears to capture a broader metabolic vulnerability, reflecting ectopic lipid deposition, systemic inflammation, and early renal hemodynamic alterations that precede overt diabetes [45,50]. This pattern supports the concept that insulin resistance–related metabolic perturbation is a common upstream pathway linking cardiometabolic dysregulation to acute renal injury, with diabetes representing one, but not the only, high-burden phenotype along this continuum [53,54].

Although cardiovascular cohorts contributed the largest body of evidence, positive associations were also observed in non-cardiovascular contexts, including infectious and critical illness settings such as sepsis-associated AKI and neurocritical care, as well as mixed ICU populations [55,56,57,58]. These findings indicate that the TyG–AKI relationship is not restricted to coronary disease or revascularization procedures, but extends to broader critical care environments where metabolic and inflammatory stressors coexist. In systemic inflammatory states like sepsis, dysregulated immunity, oxidative stress, and microcirculatory dysfunction are central drivers of renal injury [58,59]. Experimental and clinical data show that sepsis-associated AKI involves endothelial activation, mitochondrial dysfunction, and profound metabolic reprogramming of tubular cells, processes that are amplified by insulin resistance–related oxidative and nitrosative stress [59,60]. In acute brain injury and other neurocritical conditions, similar interactions between systemic inflammation, hemodynamic instability, and organ crosstalk further compromise renal perfusion and tubular integrity [56,57]. The convergence of metabolic dysfunction, oxidative stress, and inflammatory amplification may therefore represent a shared mechanistic pathway across diverse clinical conditions [60,61]. However, effect estimates in non-cardiovascular populations were derived from fewer studies and demonstrated greater variability, consistent with the broader heterogeneity and incomplete understanding of sepsis- and neurocritical-care–associated AKI [57,58]. Accordingly, while the signal appears directionally consistent, larger prospective and mechanistic studies are required to clarify the magnitude and stability of associations in these settings [58,59,60]. The present findings nevertheless align with accumulating evidence that TyG and related indices track cardio-renal-metabolic vulnerability across multiple disease trajectories, supporting TyG as a general marker of metabolic susceptibility to AKI rather than a context-restricted risk indicator [62,63,64].

From a clinical perspective, the TyG index is inexpensive, reproducible, and routinely available from standard laboratory parameters. The moderate discriminative performance observed in pooled analyses suggests that TyG should not be considered a standalone diagnostic tool for AKI. Rather, it may serve as a metabolic risk stratification marker that complements established clinical and procedural predictors. In cardiovascular practice—particularly in patients undergoing PCI, CABG, or treatment for acute coronary syndromes—incorporating metabolic risk profiling into peri-procedural assessment may help identify individuals with reduced renal resilience. Within a cardiometabolic framework, TyG could be integrated into composite risk models that account for vascular disease burden, glycemic status, and renal vulnerability.

An important point is that the TyG index should not be interpreted as a replacement for established AKI diagnostic or monitoring parameters. Dynamic deterioration in serum creatinine or eGFR, reduced urine output, and urinary protein or other urinary abnormalities remain more directly linked to the recognition of ongoing kidney injury. By contrast, TyG is more plausibly understood as a pre-injury or baseline metabolic vulnerability marker, reflecting insulin resistance, endothelial dysfunction, oxidative stress, and microvascular susceptibility. These indicators therefore serve different clinical purposes rather than functioning as interchangeable alternatives. In this context, TyG may have potential value for identifying patients at elevated AKI risk before overt renal dysfunction becomes apparent, but current evidence does not support claims that it is more sensitive than conventional renal function or urinary markers for AKI detection. Future studies should assess its added value within multivariable prediction frameworks, including discrimination, calibration, and reclassification beyond established clinical and renal biomarkers.

## 6. Strengths and Limitations

This study provides a comprehensive synthesis of available evidence, integrating categorical and continuous modeling approaches, dose–response analyses and subgroup exploration meta-analyses. The consistency across analytic strategies strengthens confidence in the robustness of the association. Several limitations merit consideration. Most included studies were observational, precluding causal inference. Residual confounding cannot be excluded despite multivariable adjustment in the original reports. Heterogeneity was moderate to substantial in certain disease categories, reflecting differences in population characteristics, exposure definitions, and adjustment models. Furthermore, variability in TyG categorization and cut-off definitions across studies may have contributed to between-study heterogeneity, and standardized exposure modeling would facilitate more precise comparisons in future research. In addition, potential publication bias was detected in the overall categorical synthesis, and trim-and-fill analysis attenuated the pooled random-effects estimate from 1.95 to 1.55. This suggests that the magnitude of association may have been overestimated in the unadjusted analysis, although the direction of association remained unchanged. An apparent discrepancy was observed between the meta-regression and subgroup analyses regarding diabetes-related heterogeneity. While study-level diabetes prevalence was associated with effect-size variation in meta-regression, subgroup analyses did not show significant interaction according to diabetic versus non-diabetic cohort classification. These findings are not directly equivalent and may differ because they reflect different levels of aggregation and different statistical power; however, the inconsistency remains unresolved and should be interpreted cautiously. Finally, while we explored diagnostic performance, limited reporting prevented detailed evaluation of calibration and incremental predictive value.

## 7. Conclusions

Elevated TyG index is associated with increased AKI risk across heterogeneous clinical settings, with the strongest evidence observed in cardiovascular populations. These findings support TyG primarily as a candidate marker of metabolic vulnerability rather than a validated stand-alone tool for early AKI risk stratification. Future studies should determine whether TyG provides incremental predictive value beyond established renal and clinical indicators, validate its performance in prospective and multicenter cohorts, and clarify how it may be incorporated into multivariable AKI risk assessment frameworks.

## Data Availability

Data sharing is not applicable to this article as no datasets were generated or analyzed during the current study.

## Supplementary Materials

The following supporting information can be downloaded at: https://www.mdpi.com/article/10.3390/jcm15093390/s1, Table S1: PRISMA 2020 Checklist; Table S2: Search strategies for Each Database; Table S3: Risk of bias assessment using the QUIPS tool; Table S4: Univariable random-effects meta-regression analyses of study-level moderators for the association between TyG index and acute kidney injury; Figure S1: Subgroup analysis in cardiovascular cohorts; Figure S2: Interaction meta-analysis (ratio of odds ratios); Figures S3: Funnel plot with trim-and-fill adjustment for publication bias; Figure S4: Funnel plots for cardiovascular subgroup analyses; Figure S5: Sensitivity analyses of the association between TyG index and AKI.
