# Supplementary material for: Triglyceride–Glucose Index and Acute Kidney Injury: A Systematic Review and Meta-Analysis
Source: J Clin Med. 2026 Apr 29;15(9):3390. doi: 10.3390/jcm15093390 (PMC13163742; doi:10.3390/jcm15093390)

Supplementary Materials

Title: The triglyceride–glucose index and risk of acute kidney injury across cardiovascular and diverse clinical populations: a systematic review and meta-analysis

Table of Contents

- Supplementary Tables
  - - Table S1. PRISMA 2020 Checklist .... Page S2-5
  - - Table S2. Search Strategy .... Page S6
  - - Table S3. QUIPS Risk-of-Bias Assessment .... Page S7-8
  - - Table S4. Meta-regression analyses .... Page S9

Supplementary Figures

- - Figure S1. Forest plot of subgroup analyses in cardiovascular cohorts .... Page S10
- - Figure S2. Interaction meta-analysis.............................. Page S11
- - Figure S3-4. Publication bias.............................. Page S12-13
- - Figure S5. Sensitivity analysis .... Page S14

Table S1: PRISMA 2020 Checklist

Section and Topic	Item #	Checklist item	Location where item is reported
**TITLE**			
Title	1	Identify the report as a systematic review.	1
**ABSTRACT**			
Abstract	2	See the PRISMA 2020 for Abstracts checklist.	2
**INTRODUCTION**			
Rationale	3	Describe the rationale for the review in the context of existing knowledge.	4
Objectives	4	Provide an explicit statement of the objective(s) or question(s) the review addresses.	4,5
**METHODS**			
Eligibility criteria	5	Specify the inclusion and exclusion criteria for the review and how studies were grouped for the syntheses.	5
Information sources	6	Specify all databases, registers, websites, organisations, reference lists and other sources searched or consulted to identify studies. Specify the date when each source was last searched or consulted.	5
Search strategy	7	Present the full search strategies for all databases, registers and websites, including any filters and limits used.	5
Selection process	8	Specify the methods used to decide whether a study met the inclusion criteria of the review, including how many reviewers screened each record and each report retrieved, whether they worked independently, and if applicable, details of automation tools used in the process.	5,6
Data collection process	9	Specify the methods used to collect data from reports, including how many reviewers collected data from each report, whether they worked independently, any processes for obtaining or confirming data from study investigators, and if applicable, details of automation tools used in the process.	6
Data items	10a	List and define all outcomes for which data were sought. Specify whether all results that were compatible with each	6

Section and Topic	Item #	Checklist item	Location where item is reported
		outcome domain in each study were sought (e.g. for all measures, time points, analyses), and if not, the methods used to decide which results to collect.	
	10b	List and define all other variables for which data were sought (e.g. participant and intervention characteristics, funding sources). Describe any assumptions made about any missing or unclear information.	6
Study risk of bias assessment	11	Specify the methods used to assess risk of bias in the included studies, including details of the tool(s) used, how many reviewers assessed each study and whether they worked independently, and if applicable, details of automation tools used in the process.	6,7
Effect measures	12	Specify for each outcome the effect measure(s) (e.g. risk ratio, mean difference) used in the synthesis or presentation of results.	6
Synthesis methods	13a	Describe the processes used to decide which studies were eligible for each synthesis (e.g. tabulating the study intervention characteristics and comparing against the planned groups for each synthesis (item #5)).	6,7
	13b	Describe any methods required to prepare the data for presentation or synthesis, such as handling of missing summary statistics, or data conversions.	6
	13c	Describe any methods used to tabulate or visually display results of individual studies and syntheses.	6
	13d	Describe any methods used to synthesize results and provide a rationale for the choice(s). If meta-analysis was performed, describe the model(s), method(s) to identify the presence and extent of statistical heterogeneity, and software package(s) used.	6
	13e	Describe any methods used to explore possible causes of heterogeneity among study results (e.g. subgroup analysis, meta-regression).	6
	13f	Describe any sensitivity analyses conducted to assess robustness of the synthesized results.	7
Reporting bias	14	Describe any methods used to assess risk of bias due to missing results in a synthesis (arising from reporting biases).	7

Section and Topic	Item #	Checklist item	Location where item is reported
assessment			
Certainty assessment	15	Describe any methods used to assess certainty (or confidence) in the body of evidence for an outcome.	6,7
**RESULTS**			
Study selection	16a	Describe the results of the search and selection process, from the number of records identified in the search to the number of studies included in the review, ideally using a flow diagram.	7
	16b	Cite studies that might appear to meet the inclusion criteria, but which were excluded, and explain why they were excluded.	7
Study characteristics	17	Cite each included study and present its characteristics.	8
Risk of bias in studies	18	Present assessments of risk of bias for each included study.	8,9
Results of individual studies	19	For all outcomes, present, for each study: (a) summary statistics for each group (where appropriate) and (b) an effect estimate and its precision (e.g. confidence/credible interval), ideally using structured tables or plots.	9,10
Results of syntheses	20a	For each synthesis, briefly summarise the characteristics and risk of bias among contributing studies.	10
	20b	Present results of all statistical syntheses conducted. If meta-analysis was done, present for each the summary estimate and its precision (e.g. confidence/credible interval) and measures of statistical heterogeneity. If comparing groups, describe the direction of the effect.	10,11,12
	20c	Present results of all investigations of possible causes of heterogeneity among study results.	12,13,14,15.16
	20d	Present results of all sensitivity analyses conducted to assess the robustness of the synthesized results.	16
Reporting biases	21	Present assessments of risk of bias due to missing results (arising from reporting biases) for each synthesis assessed.	16

Section and Topic	Item #	Checklist item	Location where item is reported
Certainty of evidence	22	Present assessments of certainty (or confidence) in the body of evidence for each outcome assessed.	10,11,12
**DISCUSSION**			
Discussion	23a	Provide a general interpretation of the results in the context of other evidence.	16,17,18
	23b	Discuss any limitations of the evidence included in the review.	19
	23c	Discuss any limitations of the review processes used.	19
	23d	Discuss implications of the results for practice, policy, and future research.	20
**OTHER INFORMATION**			
Registration and protocol	24a	Provide registration information for the review, including register name and registration number, or state that the review was not registered.	/
	24b	Indicate where the review protocol can be accessed, or state that a protocol was not prepared.	/
	24c	Describe and explain any amendments to information provided at registration or in the protocol.	/
Support	25	Describe sources of financial or non-financial support for the review, and the role of the funders or sponsors in the review.	20
Competing interests	26	Declare any competing interests of review authors.	20
Availability of data, code and other materials	27	Report which of the following are publicly available and where they can be found: template data collection forms; data extracted from included studies; data used for all analyses; analytic code; any other materials used in the review.	/

Table S2. Search strategies for Each Database

Database	Search Strategy
PubMed (MEDLINE)（n=49）	**#1 TyG index concept**<br>("triglyceride-glucose index"[tiab] OR "triglyceride glucose index"[tiab] OR "TyG index"[tiab] OR TyG[tiab])<br>**#2 Acute kidney injury concept**<br>("Acute Kidney Injury"[Mesh] OR "acute kidney injury"[tiab] OR AKI[tiab] OR "acute renal injury"[tiab] OR "acute renal failure"[tiab])<br>**#3 Combined search**<br>#1 AND #2<br>**#4 Exclusion**<br>NOT (animals[mh] NOT humans[mh])
Embase (Elsevier)<br>(n=1051)	**1. TyG index concept**<br>('triglyceride glucose index'/exp OR ("TyG" OR "TyG index" OR "triglyceride-glucose index").ti,ab,kw.)<br>**2. Acute kidney injury concept**<br>('acute kidney injury'/exp OR 'acute renal failure'/exp OR (AKI OR "acute kidney injury" OR "acute renal injury").ti,ab,kw.)<br>**3. Combined search**<br>1 AND 2<br>**4. Exclusion**<br>(exp animal/ OR nonhuman/) NOT exp human/
Web of Science Core Collection<br>(n=56)	**#1 Topic: TyG index**<br>TS=("triglyceride-glucose index" OR "triglyceride glucose index" OR "TyG index" OR TyG)<br>**#2 Topic: Acute kidney injury**<br>TS=("acute kidney injury" OR AKI OR "acute renal injury" OR "acute renal failure")<br>**#3 Combined search**<br>#1 AND #2

Table S3. Risk of bias assessment using the QUIPS tool

Study (Year)	Study participation	Study attrition	Prognostic factor measurement	Outcome measurement	Study confounding	Statistical analysis & reporting	Overall risk of bias
Qin 2020	Moderate	Low	Low	Low	Low	Low	Low
Hu 2022	Low	Moderate	Low	Low	Low	Moderate	Moderate
Yang 2023	Low	Low	Low	Low	Low	Moderate	Low
Jin 2023	Low	Low	Low	Low	Low	Moderate	Low
Zhu 2023	Moderate	Low	Low	Low	Moderate	Low	Moderate
Fang 2024	Low	Moderate	Low	Low	Low	Low	Low
Huang 2024	Low	Low	Low	Low	Moderate	Low	Low
Cai 2024	Moderate	Moderate	Low	Low	Low	Low	Low
Shi 2024	Low	Low	Low	Low	Low	Low	Low
Wang 2024	Low	Moderate	Low	Low	Low	Low	Low
Chi 2024	Moderate	Low	Low	Low	Low	Moderate	Low
He 2024	Low	Moderate	Low	Low	Moderate	Moderate	Moderate
Wang 2024	Moderate	Low	Low	Low	Low	Low	Low
Zhang 2024	Moderate	Moderate	Low	Low	Low	Low	Moderate
Zhou 2025	Low	Low	Low	Low	Low	Low	Low
Zhang 2025	Moderate	Low	Low	Low	Low	Low	Low
Zhang 2025	Low	Moderate	Low	Low	Low	Moderate	Moderate
Pan 2025	Moderate	Low	Low	Low	Low	Low	Low
Qiu 2025	Low	Moderate	Low	Low	Low	Low	Low
Hou 2025	Moderate	Moderate	Low	Low	Low	c	Low

Li 2025	Low	Moderate	Low	Low	Low	Low	Low
Yang 2025	Low	Low	Low	Low	Moderate	Moderate	Moderate
Zhang 2025	Moderate	Low	Low	Low	Low	Low	Low
Zhang 2025	Low	Moderate	Low	Low	Low	Low	Low
Jin 2025	Low	Low	Low	Low	Low	Low	Low
Wang 2025	Moderate	Low	Low	Low	Low	Moderate	Moderate
Lu 2025	Low	Low	Low	Low	Low	Low	Low
Zhang 2025	Moderate	Low	Low	Low	Low	Low	Low
Zeng 2025	Moderate	Moderate	Low	Low	Low	Low	Moderate

Table S4. Univariable random-effects meta-regression analyses of study-level moderators for the association between TyG index and acute kidney injury

Variable	β coefficient (95% CI)	OR (95% CI)†	P value	R^2 analog (%)
Age (per 1-year increase)	−0.013 (−0.034 to 0.007)	0.99 (0.97 to 1.01)	0.194	-
Male proportion (per 1% increase)	0.004 (−0.012 to 0.020)	1.00 (0.99 to 1.02)	0.622	-
**Diabetes prevalence (per 1% increase)**	**0.006 (0.001 to 0.012)**	**1.01 (1.00 to 1.01)**	**0.021**	**50**
Publication year	−0.080 (−0.198 to 0.037)	0.92 (0.82 to 1.04)	0.168	-
Prospective vs retrospective	0.278 (−0.255 to 0.810)	1.32 (0.78 to 2.25)	0.289	-
Disease category: Metabolic/other vs Cardiovascular	0.051 (−0.382 to 0.483)	1.05 (0.68 to 1.62)	0.808	-
Disease category: Infectious/inflammatory vs Cardiovascular	0.117 (−0.449 to 0.682)	1.12 (0.64 to 1.98)	0.670	-
Disease category: Neurological/critical illness vs Cardiovascular	0.218 (−0.221 to 0.658)	1.24 (0.80 to 1.93)	0.310	-

β coefficients are presented on the log(OR) scale.

OR values represent exp(β) and indicate the multiplicative change in pooled odds ratio per unit increase in the moderator.

R^2 analog reflects the proportion of between-study heterogeneity explained by each covariate, calculated from the reduction in τ^2 relative to the null model.

All analyses were performed using restricted maximum likelihood estimation under a random-effects framework.

Figure S1. Subgroup analysis in cardiovascular cohorts

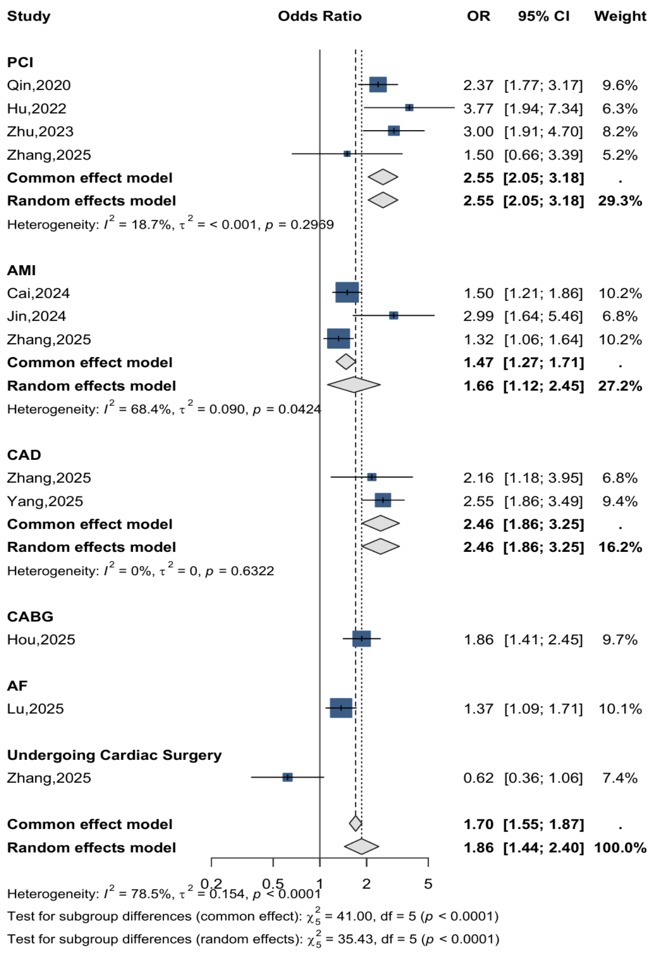

Figure S2. Interaction meta-analysis (ratio of odds ratios)

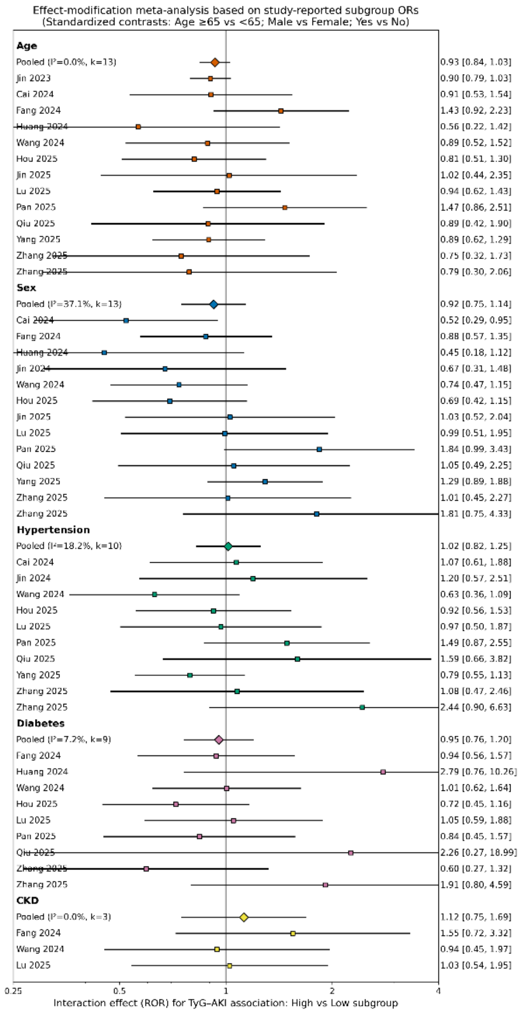

Figure S3. Funnel plot with trim-and-fill adjustment for publication bias

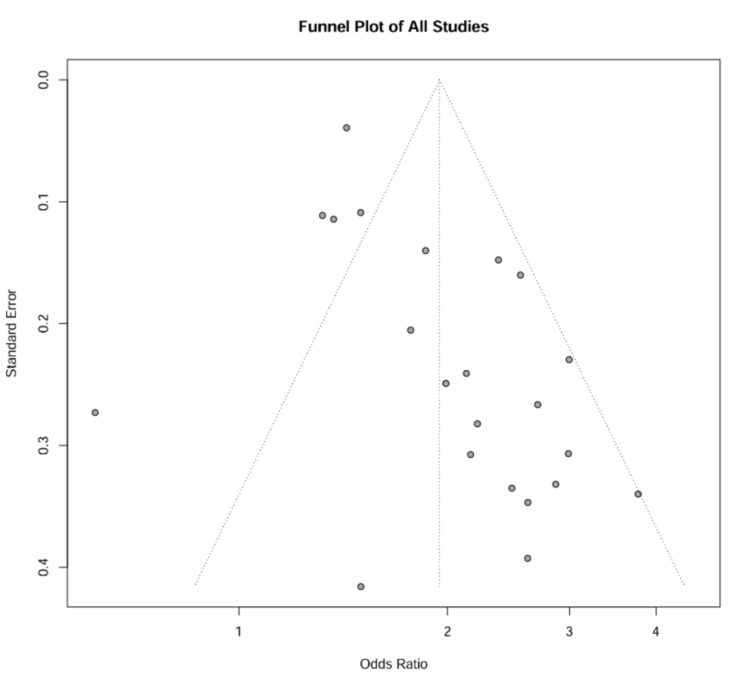

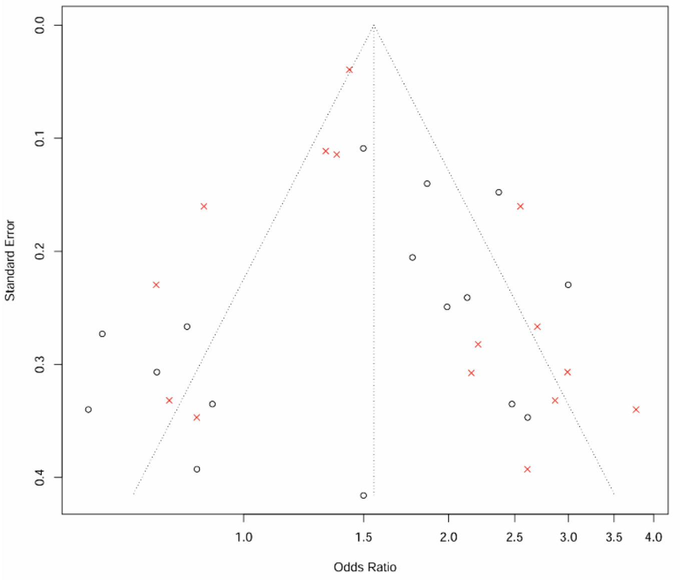

Figure S4. Funnel plots for cardiovascular subgroup analyses

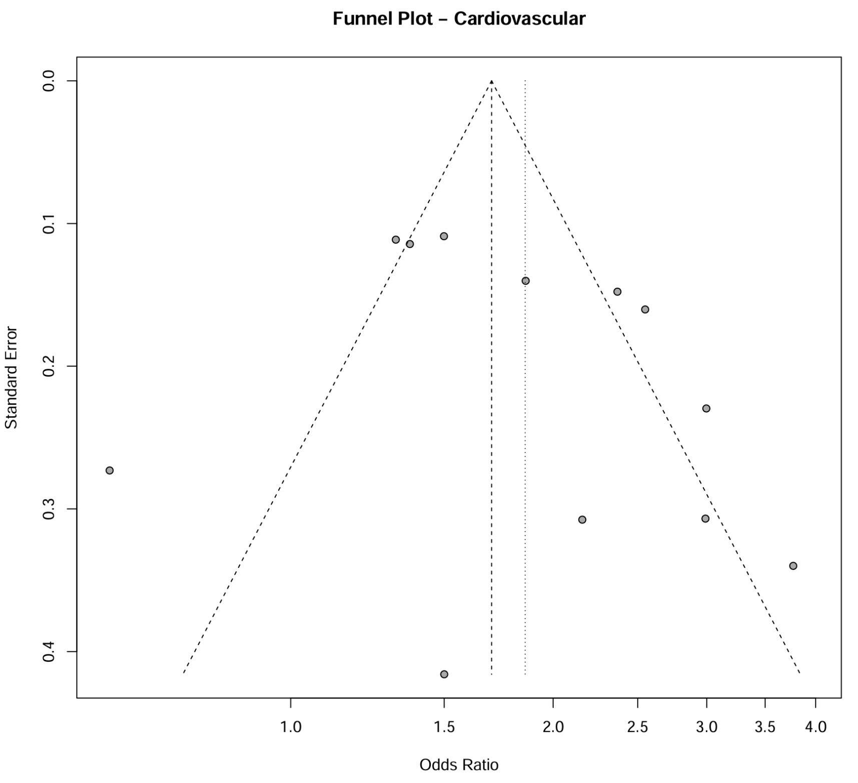

Figure S5. Sensitivity analyses of the association between TyG index and AKI

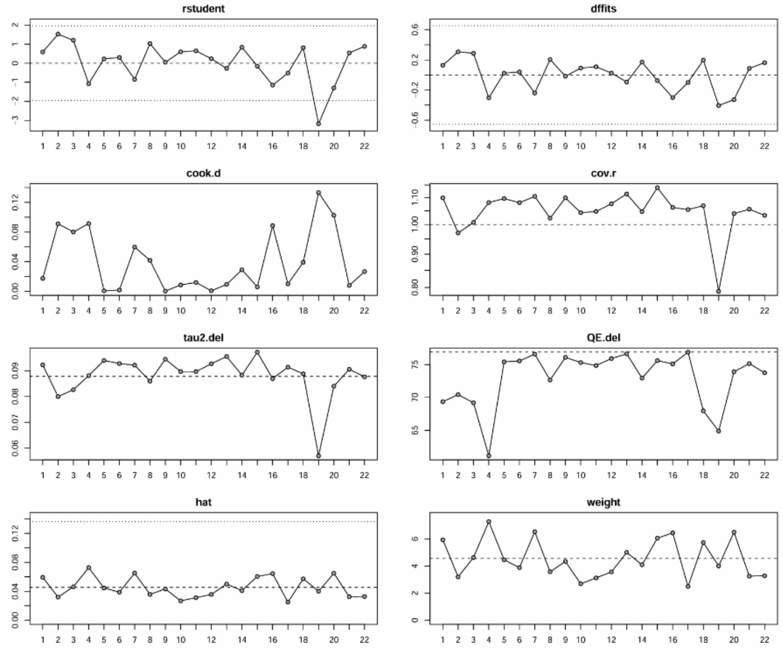

Study	P−value	Tau2	Tau	I2	OR	95%−CI
Omitting Qin,2020	< 0.0001	0.0922	0.3037	71.2%	1.93	[1.63; 2.28]
Omitting Hu,2022	< 0.0001	0.0800	0.2828	71.6%	1.90	[1.63; 2.22]
Omitting Zhu,2023	< 0.0001	0.0826	0.2875	71.1%	1.90	[1.63; 2.23]
Omitting Jin,2023	< 0.0001	0.0881	0.2968	67.3%	2.00	[1.69; 2.35]
Omitting Fang,2024	< 0.0001	0.0939	0.3065	73.5%	1.94	[1.65; 2.29]
Omitting Huang,2024	< 0.0001	0.0928	0.3046	73.5%	1.94	[1.65; 2.29]
Omitting Cai,2024	< 0.0001	0.0921	0.3035	73.9%	1.99	[1.68; 2.35]
Omitting Jin,2024	< 0.0001	0.0859	0.2932	72.5%	1.92	[1.63; 2.25]
Omitting Wang,2024	< 0.0001	0.0945	0.3074	73.7%	1.95	[1.65; 2.30]
Omitting He,2024	< 0.0001	0.0896	0.2994	73.4%	1.93	[1.65; 2.27]
Omitting Chi,2024	< 0.0001	0.0896	0.2994	73.3%	1.93	[1.64; 2.27]
Omitting Zhang,2025	< 0.0001	0.0927	0.3044	73.7%	1.94	[1.65; 2.29]
Omitting Pan,2025	< 0.0001	0.0955	0.3090	73.9%	1.96	[1.66; 2.32]
Omitting Qiu,2025	< 0.0001	0.0883	0.2971	72.6%	1.92	[1.64; 2.26]
Omitting Hou,2025	< 0.0001	0.0972	0.3117	73.6%	1.96	[1.66; 2.32]
Omitting Lu,2025	< 0.0001	0.0869	0.2948	73.4%	2.00	[1.70; 2.35]
Omitting Zhang,2025	< 0.0001	0.0914	0.3023	74.0%	1.96	[1.67; 2.31]
Omitting Yang,2025	< 0.0001	0.0888	0.2980	70.6%	1.92	[1.63; 2.26]
Omitting Zhang,2025	< 0.0001	0.0571	0.2389	69.2%	2.01	[1.74; 2.31]
Omitting Zhang,2025	< 0.0001	0.0840	0.2898	72.9%	2.00	[1.70; 2.35]
Omitting Jin,2025	< 0.0001	0.0905	0.3009	73.4%	1.93	[1.64; 2.28]
Omitting Zeng,2025	< 0.0001	0.0876	0.2960	72.9%	1.92	[1.64; 2.26]
**Total (95% CI)**	**< 0.0001**	**0.0878**	**0.2963**	**72.7%**	**1.95**	**[1.66; 2.28]**

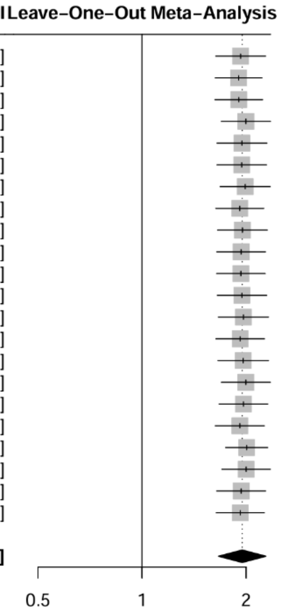

Supplement: Supplementary file 1 [file jcm-15-03390-s001.zip › jcm-4220759-supplementary.pdf]
